# Supplementary material for: Between-subject variability in the influence of mental imagery on conscious perception
Source: Sci Rep. 2019 Oct 30;9:15658. doi: 10.1038/s41598-019-52072-1 (PMC6821778; doi:10.1038/s41598-019-52072-1)
Supplement: Supplementary file 1 — Supplementary Material [file 41598_2019_52072_MOESM1_ESM.pdf]

Supplementary material for: ‘Between-subject variability in the influence of mental imagery on conscious perception’

N. Dijkstra, M. Hinne, S.E. Bosch and M.A.J. van Gerven

1 Power calculation

We performed a power-calculation using G-power (Erdfelder et al., 2009) to determine the required number of participants needed to investigate individual differences. Previous research investigating individual differences in imagerys effect of binocular rivalry have reported a range of correlation strengths: 0.38 (Bergmann et al., 2015), 0.57 (Keogh and Pearson, 2014), 0.45 (Keogh et al., 2016) and 0.73 (Pearson et al., 2011). Assuming a correlation of 0.35, a power of 0.8, an alpha of 0.05 and a two-tailed test, the power calculation showed that we required a sample size of 59.

2 Bayesian hierarchical model

2.1 Model definition

The model is based on the psychometric curve by Wichmann and Hill (2001). It defines the response function as

$$f(x; g, l, u, v) = g + (1 - g - l)\Phi\left(\frac{x - u}{v}\right) \quad , \tag{1}$$

in which x is the manipulated contrast, $g \in [0, 1]$ is the *guess rate*, $l \in [0, 1]$ the *lapse rate*, $u \in [0, 1]$ the *bias*, $v \in \mathbb{R}$ the *discrimination sensitivity* and $\Phi(\cdot)$ the cumulative Normal distribution. For examples of such curves, we defer to the main document. From this definition we construct a Bayesian hierarchical model as follows. For participant $i = 1, \ldots, N$, condition $k \in \{1, 2\}$ and observed stimulus contrast level $j = 1, \ldots, M$ we define the likelihood

$$\begin{aligned} \mu_{ijk} \mid g_{ik}, l_{ik}, u_{ik}, v_{ik}, c_j &= f(c_j; g_{ik}, l_{ik}, u_{ik}, v_{ik}) \\ d_{ijk} &\sim \text{Normal}\left(\mu_{ijk}, \tau_i\right) \quad . \end{aligned} \tag{2}$$

Here, the parameter τ_i is the precision of subject i, corresponding to the inverse of its noise level. This term is estimated by placing a vague prior distribution over it, that is

$$\tau_i \mid \alpha_\tau, \beta_\tau \sim \text{Gamma}\left(\alpha_\tau, \beta_\tau\right) \tag{3}$$

with $\alpha_\tau = \beta_\tau = 0.001$.

Hierarchy is introduced to the model in a standard fashion by centering the prior distribution for each participant-level parameter on the corresponding population-level parameter. That is,

$$\begin{aligned} g_{ik} \mid \hat{g}_k, \tau_g &\sim \text{TNormal}_0^1\left(\hat{g}, \tau_g\right) \\ l_{ik} \mid \hat{g}_k, \tau_l &\sim \text{TNormal}_0^1\left(\hat{l}, \tau_l\right) \\ u_{ik} \mid \hat{u}_k, \tau_u &\sim \text{TNormal}_0^1\left(\hat{u}, \tau_u\right) \\ v_{ik} \mid \hat{v}_k, \tau_v &\sim \text{Gamma}\left(\hat{v}^2\tau_v, \hat{v}\tau_v\right) \quad , \end{aligned} \tag{4}$$

where $\text{TNormal}_a^b(\mu, \tau)$ is the Normal distribution with mean μ and precision τ, but with its domain truncated to the interval $[a, b]$.

For the population-level parameters (indicated with the ˆ symbol) we define (hyper-)priors in which we encode our informed prior beliefs on the psychometric curve. We define

$$\begin{aligned} \hat{g}_k \mid \mu_{\hat{g}}, \tau_{\hat{g}} &\sim \text{TNormal}_0^1(\mu_{\hat{g}}, \tau_{\hat{g}}) \\ \hat{l}_k \mid \mu_{\hat{l}}, \tau_{\hat{l}} &\sim \text{TNormal}_0^1(\mu_{\hat{l}}, \tau_{\hat{l}}) \\ \hat{u}_k \mid \mu_{\hat{u}}, \tau_{\hat{u}} &\sim \text{TNormal}_0^1(\mu_{\hat{u}}, \tau_{\hat{u}}) \\ \hat{v}_k \mid \mu_{\hat{v}}, \tau_{\hat{v}} &\sim \text{Gamma}(\mu_{\hat{v}}^2 \tau_{\hat{v}}, \mu_{\hat{v}} \tau_{\hat{v}}) \ , \end{aligned} \tag{5}$$

where we set $\mu_{\hat{g}} = 0.02$, $\tau_{\hat{g}} = \tau_{\hat{l}} = \tau_{\hat{v}} = 1/0.1^2$, $\mu_{\hat{l}} = \mu_{\hat{v}} = 0.1$, $\mu_{\hat{u}} = 0.5$ and $\tau_{\hat{u}} = 1/0.5^2$, representing the expected group-level curves.[1]. We set the prior on the guess rate higher than the lapse rate because it is more difficult to reach 100 percent dominance while the other stimulus is fixed at 0.4 contrast than it is to reach 0 percent dominance (for more details, see main text).

Similarly, we estimate the precision parameters that determine the amount of variability of participants around the group-level means. As we have no strong prior beliefs about this variability, we use vague priors (Lee and Vanpaemel, 2018), defined via

$$\begin{aligned} \tau_g \mid \alpha_{\tau_g}, \beta_{\tau_g} &\sim \text{Gamma}(\alpha_{\tau_g}, \beta_{\tau_g}) \\ \tau_l \mid \alpha_{\tau_l}, \beta_{\tau_l} &\sim \text{Gamma}(\alpha_{\tau_l}, \beta_{\tau_l}) \\ \tau_u \mid \alpha_{\tau_u}, \beta_{\tau_u} &\sim \text{Gamma}(\alpha_{\tau_u}, \beta_{\tau_u}) \\ \tau_v \mid \alpha_{\tau_v}, \beta_{\tau_v} &\sim \text{Gamma}(\alpha_{\tau_v}, \beta_{\tau_v}) \ , \end{aligned} \tag{6}$$

where we set $\alpha_m = \beta_m = 0.001$ for $m \in \{\tau_g, \tau_l, \tau_u, \tau_v, \tau_i\}$.

2.2 Inference

Inference of the model is done via Gibbs Markov chain Monte Carlo sampling, using the JAGS software (Plummer, 2003). We computed 4 parallel chains of 100 000 samples and verified convergence of the sampler visually as well as via computing the potential scale reduction factor $\hat{R}$ for each parameter, and asserting that its value remained below the common heuristic of 1.1 (Brooks and Gelman, 1997). This procedure results in the approximated posterior distribution $p(\boldsymbol{\theta} \mid D, c, \boldsymbol{\psi})$ where $\boldsymbol{\theta}$ contains all latent parameters, $D \in [0,1]^{M \times N \times K}$ is the observed indicated dominance, $c \in [0,1]^M$ is the manipulated contrast and $\boldsymbol{\psi}$ is the set of hyperparameters of the model.

2.3 Model comparison using Bayes factors

The approximated posterior distribution of the latent parameters implies a distribution over the differences of these parameters. Consider for example the group-level bias term. We have available the posterior distribution of $\hat{u}_k$ for both conditions, so we can derive the implied posterior distribution over $\hat{\delta}_u = \hat{u}_2 - \hat{u}_1$. Subsequently, we test whether $\hat{\delta}_u \neq 0$, that is we compare the alternative hypothesis $H_+ : \hat{\delta}_u \neq 0$ with the null model in which there is no effect, that is, $H_0 : \hat{\delta}_u \neq 0$.

As H_0 is a special case of the full model, we can use the Savage-Dickey method to conveniently compute the corresponding Bayes factors for these tests (Dickey, 1971; Wagenmakers et al., 2010). For this method, we approximate both the posterior as well as the prior model (that is, $p(\theta \mid D, c, \boldsymbol{\psi})$ and $p(\theta \mid c, \boldsymbol{\psi})$), and compute the ratio of these densities at $\hat{\delta}_u = 0$, i.e.:

$$BF_{+0}^{\hat{\delta}_u} = \frac{p(D \mid H_+, \boldsymbol{\psi})}{p(D \mid H_0, \boldsymbol{\psi})} = \frac{p(\hat{\delta}_u = 0 \mid H_+, c, \boldsymbol{\psi})}{p(\hat{\delta}_u = 0 \mid H_+, D, c, \boldsymbol{\psi})} \ . \tag{7}$$

The Bayes factors for the different comparisons, both at the group-level and the participant-level, are shown in the main text.

2.4 Robustness check

To check that our results were not too influenced by our choices of hyperparameter settings, we ran the analysis again, once with broader priors and once with narrower priors. The results are shown

[1] Note that it is typically easier to think of standard deviation and variance rather than precision. Hence we transform our beliefs about standard deviation into beliefs about precision via the identity $\tau = 1/\sigma^2$.

in Figure S1. The pattern of Bayes factors using a wide or narrow prior is very similar to that observed using the original prior. The correlations between the log(BFs) is shown in Figure S2. All correlations are higher than 0.94, indicating that differences in the prior did not substantially change our findings.

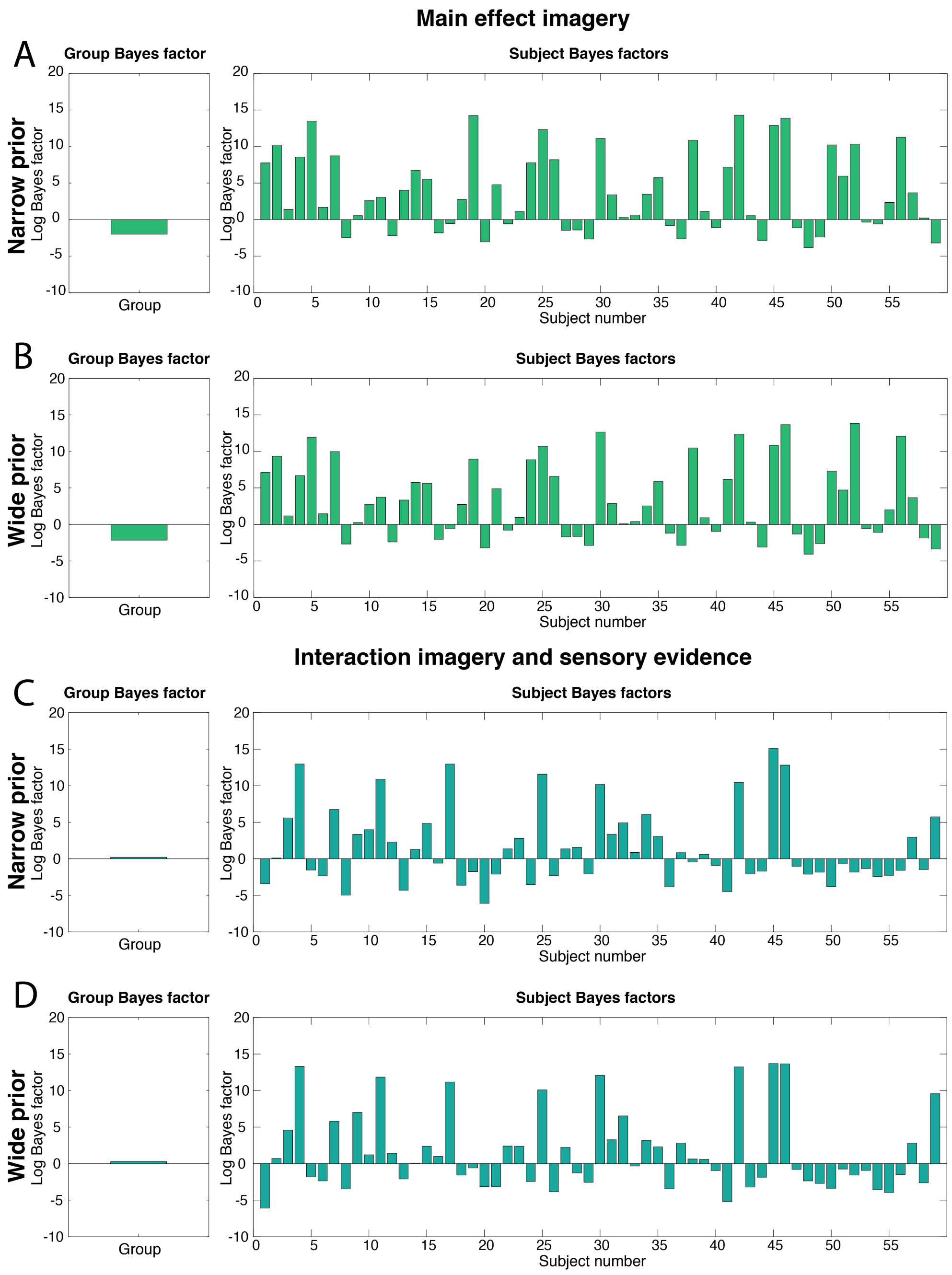

Figure S1: **Bayes factors robustness check: Bayes factors.** Bayes factors with narrow priors and with wide priors. (A) BFs for the difference in bias with narrow priors. (B) BFs for the difference in bias with wide priors. (C) BFs for the difference in discrimination sensitivity with narrow priors. (D) BFs for the difference in discrimination sensitivity with wide priors.

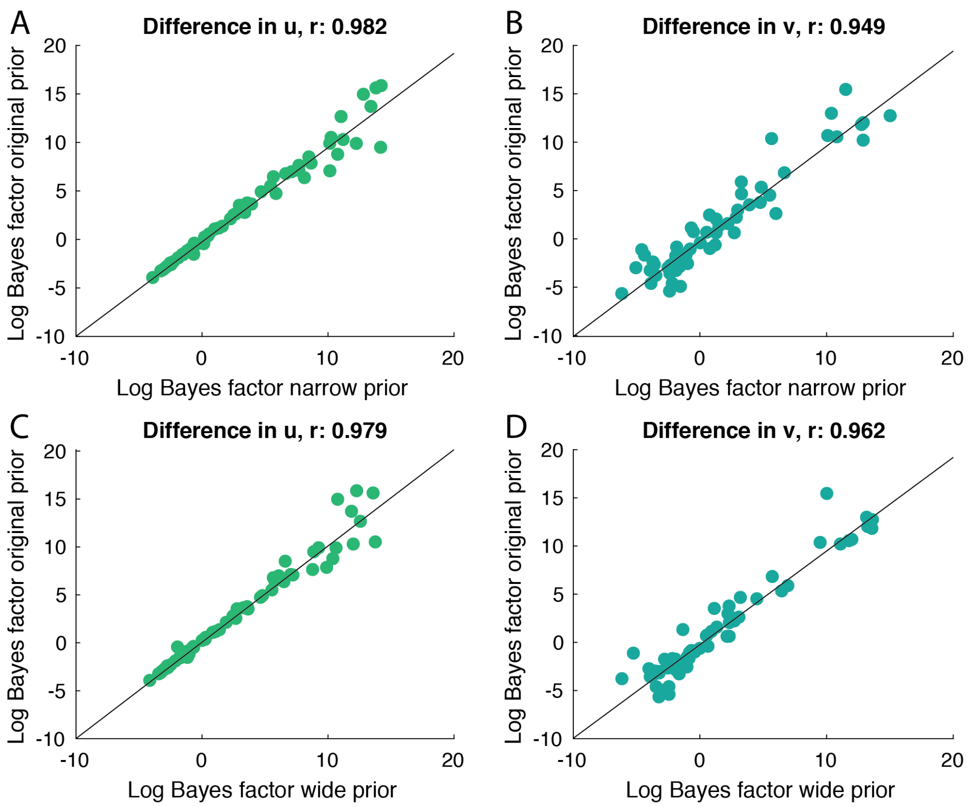

Figure S2: **Bayes factors robustness check: correlations.** Comparison between BFs using the original priors reported in the main text and using narrower (A, C) or wider (B, D) priors. Each circle represents a participant.

3 Eye dominance

Variation in eye dominance could have influenced our results. A strong eye dominance means that the subject has a higher probability of perceiving the stimulus coming in to the dominant eye compared to the other stimulus. This would result in a shift of the psychometric response function; to the left if the eye corresponding to the manipulated stimulus is dominant and to the right if the other eye is dominant. Below is illustrated what this effect would look like for very strong eye-dominance (Fig S3. This also illustrates our motivation to manipulate the stimulus associated with the dominant eye; otherwise we might not be able to estimate the full psychometric curve (Fig S3; right panel). However, the Miles test that we used to asses dominance test that we used might not have accurately captured the eye dominance relevant to the binocular rivalry task (Ding et al., 2018). Furthermore, also in the case of extreme eye dominance of the manipulated stimulus (Fig S3; middle panel), the curve might become too steep to measure any differences between conditions. This means that strong eye-dominance might have caused us to underestimate the size of the effect in some participants.

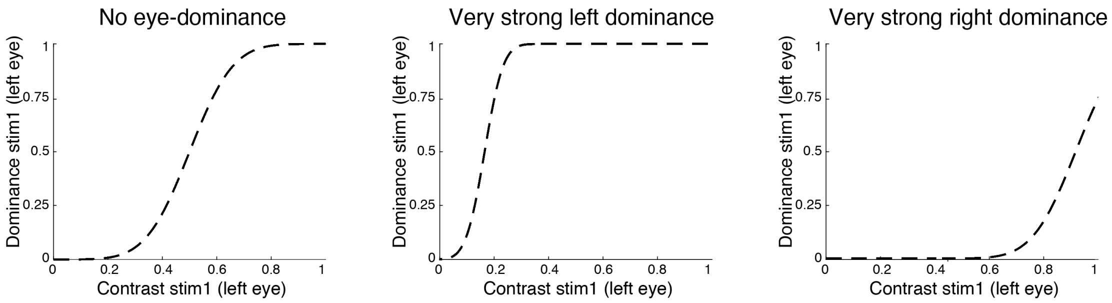

Figure S3: **Effect of strong eye-dominance on psychometric curves.** Illustration of how strong eye-dominance can influence the shape of the psychometric curve. Left panel: no eye-dominance. Middle panel: a strong dominance of the eye corresponding to the manipulated stimulus results in a shift towards the left, with low contrast values already corresponding to high dominance. Right panel: a strong dominance of the eye corresponding to the fixed stimulus results in a shift towards the right, with even high contrast no resulting in full dominance.

Importantly, it is unlikely that eye-dominance has had any influence on the direction of the effect, since we do not expect mental imagery to change biases in eye-dominance. If, however, there would be some unpredicted interaction between eye-dominance and the direction of the imagery effect, we would expect to find a relationship between the direction of the effect and eye-dominance. As illustrated in Figure S3, eye-dominance leads to a shift in the offset of the psychometric curve. Therefore, to investigate whether there is a principled relationship between eye-dominance and the direction of the effect in our data, below we plot the mean offset over conditions (representing the eye-dominance) versus the direction of the effects (Fig S4). A few things stand out. First, we have more participants on the left side of the figures. This means that more participants had an eye-dominance corresponding to the manipulated stimulus, which is in line with what we tried to achieve with the Miles test. Furthermore, the size of the main effect of increases when the mean offset is closer to 0.5, which corresponds to no eye-dominance. Finally, the number of participants showing a priming/sensitization (positive difference; above the line) versus an adaption/desensitization effect (below the line), does not change systematically for different offset values. This confirms that the direction of the imagery effect is indeed independent of eye-dominance, whereas the size of the effect can be influenced by eye-dominance. Together, this indicates that the results reported here reflect a lower bound on the size of the imagery effects.

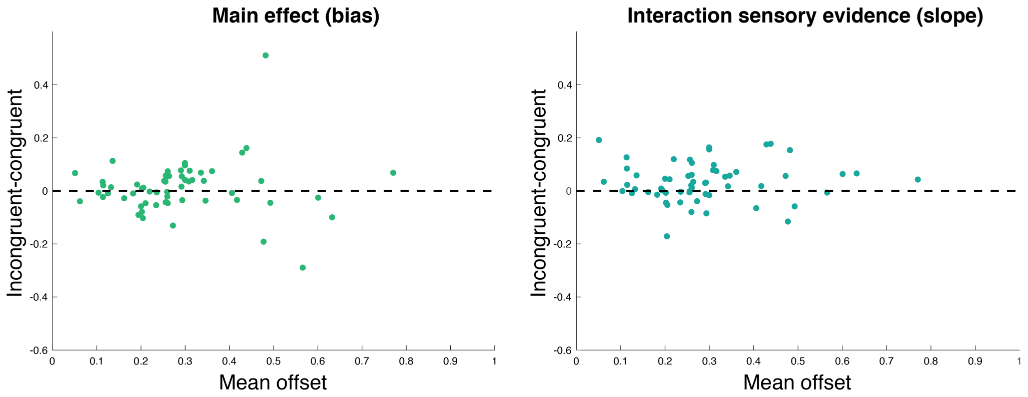

Figure S4: **Influence of eye-dominance on imagery effects.** The mean offset of the psychometric curve as an indication of eye-dominance is plotted on the x-axis. Left panel: the main effect of imagery. Right panel: interaction between imagery and sensory evidence.

References

Bergmann, J., Genç, E., Kohler, A., Singer, W., and Pearson, J. (2015). Smaller primary visual cortex is associated with stronger, but less precise mental imagery. *Cerebral cortex*, 26(9):3838–3850.

Brooks, S. P. and Gelman, A. (1997). General methods for monitoring convergence of iterative simulations. *Journal of Computational and Graphical Statistics*, 7:434–455.

Dickey, J. M. (1971). The weighted likelihood ratio, linear hypotheses on normal location parameters. *The Annals of Mathematical Statistics*, 42:204–223.

Ding, Y., Naber, M., Gayet, S., Van der Stigchel, S., and Paffen, C. L. (2018). Assessing the generalizability of eye dominance across binocular rivalry, onset rivalry, and continuous flash suppression. *Journal of Vision*, 18(6):6.

Erdfelder, E., Faul, F., Buchner, A., and Lang, A.-G. (2009). Statistical power analyses using G*Power 3.1: test for correlation and regression analyses. *Behavioral Research Methods*, 41(4):1149–1160.

Keogh, R., Bergmann, J., and Pearson, J. (2016). Cortical excitability modulates the sensory strength of visual mental imagery. *bioarxiv*.

Keogh, R. and Pearson, J. (2014). The sensory strength of voluntary visual imagery predicts visual working memory capacity. *Journal of Vision*, 14(12):1427–1431.

Lee, M. D. and Vanpaemel, W. (2018). Determining informative priors for cognitive models. *Psychonomic Bulletin & Review*, 25(1):114–127.

Pearson, J., Rademaker, R. L., and Tong, F. (2011). Evaluating the mind's eye: The metacognition of visual imagery. *Psychological Science*, 22(12):1535–1542.

Plummer, M. (2003). JAGS: A program for analysis of Bayesian graphical models using Gibbs sampling.

Wagenmakers, E. J., Lodewyckx, T., Kuriyal, H., and Grasman, R. (2010). Bayesian hypothesis testing for psychologists: A tutorial on the Savage-Dickey method. *Cognitive Psychology*, 60(3):158–189.

Wichmann, F. A. and Hill, N. J. (2001). The psychometric function: I. fitting, sampling, and goodness of fit. *Perception & Psychophysics*, 63(8):1293–1313.
